# Collapse of Better Place: A Managerial Cognition Perspective on the Failure of an Entrepreneurial Initiative

**DOI:** 10.3389/fpsyg.2022.846434

**Published:** 2022-06-15

**Authors:** Mei-Hsing Lin, Hsin-Hui Chou

**Affiliations:** Department of Business Administration, National Cheng Kung University, Tainan, Taiwan

**Keywords:** business model, managerial cognition, entrepreneurial failure, value creation, value capture, case study

## Abstract

The survival of any entrepreneurial initiative depends on a working business model that could create value for the customers and, simultaneously, allow the firm to capture value from what has been created. Despite increased attention to business model research, the understanding of business models’ impact on entrepreneurial development is quite constrained. In particular, the question of how an entrepreneurial firm’s business model is influenced by its organizational members’ managerial cognition remains under-explored. To tackle this research question, we drew a linkage between the business model literature and a managerial cognition perspective to build the theoretical foundation. We used this theoretical lens to investigate the failure of Better Place, an Israeli entrepreneurial company that focused on its proprietary battery-swap electric vehicles. In our findings, we argued that organizational members’ managerial-cognition-based conceptual framework is critical to the business model decision-making of an entrepreneurial firm. The discrepant and strongly held conceptual framework may result in misjudgment of environmental changes, especially when emerging-market numbers in an industry are high, and consensus regarding technology innovation in an industry is still lacking. The improper conceptual framework can generate mistaken business models, which further bring about an organizational decline. It is crucial for entrepreneurial firms to learn and improve existing conceptual frameworks and consequent business models by business interaction in the initiative stage if they are to avoid failure. The research outcome paves the way for future empirical studies to contribute to machine learning in the field of cognitive automation, artificial-intelligence-driven smart manufacturing, and sustainable industrial value creation in the era of digital transformation.

## Introduction

Entrepreneurs usually adopt creative ways to seek solutions to customer problems, through which lucrative gains can be realized in business ventures (Ries, 2011; Blank, 2013). This venturing process is characterized by boundary-spanning resource bricolage and mobilization (Reypens et al., 2021) and trial and error (Blank and Dorf, 2012), aiming to develop a business model that could strike an adequate balance between value creation and value capture (Morris et al., 2005; Sarooghi et al., 2021). From a value-based perspective (Brandenburger and Stuart, 1996), value is a central component in determining an organization’s survival and prosperity, including entrepreneurial firms, within which value could be jointly created at different levels while the created value needs to be captured using competition or isolating mechanisms (Lepak et al., 2007).

The development of business models for entrepreneurial initiatives has become an important issue for both researchers and practitioners (Zott and Amit, 2007; George and Bock, 2011; Del Bosco et al., 2019), along with the emerging trend of business model research within the fields of strategy (Wirtz et al., 2016; Foss and Saebi, 2017; Massa et al., 2017) and marketing (Mason and Spring, 2011; Wieland et al., 2017). However, inconsistent definitions and varied conceptualizations constrain our understanding of business models in entrepreneurial contexts. For example, Zott and Amit (2007) considered business models to be activity systems and argued that their design in terms of efficiency and novelty would impact the result of a firm’s entrepreneurial initiative, while other researchers pay particular attention to the fit between and among business model elements (Morris et al., 2005; Blank and Dorf, 2012), including customer needs, enterprises’ capabilities, and partnerships. Recently, entrepreneurship is seen from a design science view that emphasizes the identification and modifications of artifacts which could lead to a business model that maintains a balance between customer desirability, technology feasibility, and business viability (Ding, 2021; Sarooghi et al., 2021).

With the escalation of interest in business model innovation (Foss and Saebi, 2017; Massa et al., 2017; Lanzolla and Markides, 2021), a cognitive perspective has emerged as an important theoretical lens to look at the development of a business model mainly because past research devotes great efforts to examine exogenous drivers, leaving the managerial cognition under-explored. Interestingly, business models as cognitive schemas are important, yet less investigated to explain an entrepreneurial initiative’s success or failure, particularly the latter. This importance lies in which entrepreneurs make sense of opportunities and their environments to take venturing actions (Hill and Levenhagen, 1995; Nicholson and Anderson, 2005), and that entrepreneurs are reflective practitioners (Schön, 1983), whose agency in reflection would drive the pivoting of their business models (Blank, 2013). Due to the need to expand the knowledge of business models as cognitive schemas, we raised this research question as follows: how is an entrepreneurial firm’s business model influenced by its organizational members’ managerial cognition?

We formed a theoretical lens by combining a managerial cognitive perspective with business model literature and further investigated our research question of how an entrepreneurial firm’s business model is influenced by its organizational members’ managerial cognition through a case study. The case under study was Better Place, an Israeli entrepreneurial firm attempting to promote electric vehicles (EVs) based on its proprietary battery-swap system. Better Place, which was founded in 2007 and filed for bankruptcy in 2013, served as a suitable case because it had a complete developmental process for exploring the research question and because sufficient archival data were available to build the case, including the entrepreneur’s interviews and speeches in the media. The structure of the rest of this study is as follows: after presenting our theoretical foundation, we explained the rationale for adopting a qualitative case study method, then, and we presented our case findings, followed by a discussion section, an implication section, and the conclusion. A cognition perspective that focuses on managers’ mental representations, recognized as schemas (Massa et al., 2017), enables us to provide a deep understanding of the failure of an entrepreneurial business model, complementing the knowledge of business models that take from a rational design and an evolutionary learning perspective (Chesbrough, 2010; Zott and Amit, 2010). The research outcome can also serve as a foundation for future empirical studies that could contribute to cognitive automation, artificial-intelligence-driven smart manufacturing, and sustainable industrial value creation in the era of digital transformation.

## Literature Review

### An Overview of Business Models

Business models have not only attracted increased attention from business practitioners but also sparked extensive discussions among scholars and researchers. A business model depicts how an enterprise operates in a certain institutional environment (Magretta, 2002), through which the value that underpins the enterprise’s survival and prosperity could be created and captured (Teece, 2010). Thus, the development of a business model concentrates on which group of customers to serve by providing what specific offerings can be delivered at an appropriate cost, allowing profits to be generated (Osterwalder and Pigneur, 2010). That is to say, the success of a business model hinges on four interrelated elements, namely, key resources, key process, profit formula, and customer value proposition that center around a customer value proposition (Johnson et al., 2008). Seeing business models as realized strategies (Casadesus-Masanell and Ricart, 2010), business model innovation is crucially important for both existing and entrepreneurial firms to gain a strategic advantage in the face of the modern economy (Del Bosco et al., 2019; Abhishek et al., 2021) and even during the pandemic (Crick and Crick, 2020).

Following the study by Timmers (1998), research on business models continues to grow (Lanzolla and Markides, 2021). However, the definitions and conceptualizations of business models remain divergent in extant literature (Wirtz et al., 2016; Foss and Saebi, 2017; Massa et al., 2017). Although a business model is regarded as a cost-and-effectiveness structure (Casadesus-Masanell and Ricart, 2010) and a value-based architecture (Teece, 2010) that describes the functioning of a profit-seeking organization (Magretta, 2002), there are different views on its attainment. To design a feasible and viable business model, both Johnson et al. (2008) and Osterwalder and Pigneur (2010) have paid close attention to the alignment of business model constituting elements (including resources, activities, partners, and channels), while Zott and Amit (2010) have conceived of a business model as a system of interdependent and boundary-spanning activities that should render important characteristics, including operational efficiency, lock-in effects, complementary products/services, and/or novel process. Additionally, the work of conceptualizing business models has spawned mainly in the domain of strategy (DaSilva and Trkman, 2014; Lanzolla and Markides, 2021), leaving it under-developed in other areas, such as marketing. As Wieland et al. (2017) have emphasized that more efforts should be devoted to advance the understanding and conceptualization of business models in the market, and therefore, they further this movement by infusing the notion of service-dominant logic, which is based on Vargo and Lusch (2016) who stressed that the existence of a firm is to make use of its knowledge and skills to provide a good which facilitates the creation of the customer’s own value in a usage context.

It appears that the existing studies of business models tend to overweight the firm-centric resources and activities and downplay the necessary business interaction, which permits the creation and capturing of value in collective actions (Håkansson and Ford, 2002; Kohtamäki and Rajala, 2016). Mason and Spring (2011) have indicated that the business model of an enterprise is closely connected with that of its stakeholders. In other words, the development and execution of one side usually affect that of the other side, which means that enterprises can hardly have business models designed on their own. In a similar vein, Ballantyne et al. (2011) argued that the formulation of a value proposition, a central element of a business model, is not solely performed by the firm itself, but rather through the engagement with its key stakeholders, such as suppliers or customers. Particularly in the coming age of platforms and ecosystems (Jacobides et al., 2018), developing a suitable business model accordingly needs to consider this interactive nature so as to take advantage of interdependence spanning organizational boundaries (Chandna and Salimath, 2018).

### A Managerial Cognition Perspective on Business Models

Abundant research on business models has appeared in the scholarly literature over the past decades, within which branches of different thinking are developed. While Martins et al. (2015) have identified three major perspectives being employed in business model research that include rational design, evolutionary learning, and managerial cognition views, Massa et al. (2017) have classified the existing research findings into three interpretations that, respectively, see a business model as empirically grounded reality, managers’ mental representations, and a conceptual framework which aims at creating competitive advantage underpinning a firm’s survival and prosperity. Nevertheless, both of which have pointed out that the interest in a cognition perspective on business model development has escalated in research. It is fair to say that this emerging trend just reflects a social construction perspective that sees organization behaviors, including the formulation of a business model, as organization members’ conversations, interpretations, actions, and reactions occurring in day-to-day social interaction (Bleicher, 1980; Weick, 1995).

Although the rapidly changing environment urges the firms to innovate or redesign their business model, increased attention has been shifted to an endogenous look at managers’ mental representations (Martins et al., 2015; Massa et al., 2017; Frankenberger and Sauer, 2019), which affect the design components and their interrelations that constitute a business model. “Business models as managerial schemas” can be seen as a cognition turn in research, which is in parallel development with cognitive perspective in strategy (Gavetti and Rivkin, 2007; Narayanan et al., 2011). A central notion of the cognition perspective concerns that the managers in organizations grasp their surroundings and frame issues based on their individual understanding, knowledge, and experience (Weick, 1995), and thus, their decision-making and strategic actions are conditioned by their interpretations of the world around them (Gavetti and Rivkin, 2007). Based on Martins et al. (2015), business model schemas are seen as cognitive structures that could produce managerial understandings which facilitate the organization of boundary-spanning activities and resources, enabling a firm’s creation and capturing of value through managing the architecture of interdependencies. That is, the managerial schemas represent a firm’s value creation logic that organizes and structures the content of activities constituting a business model (Zott and Amit, 2010), in order to seize the perceived opportunities (Weick, 1995; Chesbrough, 2010).

Interestingly, Martins et al. (2015) further proposed two strategic processes of schema generation that facilitate the formulation and architecting of a business model; they are *analogic reasoning* and *conceptual combination*. A common ground between the two processes lies in which managers can employ existing knowledge, abstractions, and concepts from a familiar area and apply them to a new area to make sense of novelty and ideate important elements for developing a business model. In a similar vein, the managers, as well as entrepreneurs, are seen as reflective practitioners whose efforts to “make it work” just reflect their perceptions of the world and experience of past actions (Schön, 1983). Such analogic or imagining practices have been deemed a critical approach for entrepreneurs to put their initiatives into engagement with real-life circumstances (Cornelissen and Clarke, 2010; Sergeeva et al., 2021). An example of analogic reasoning practices is vividly demonstrated by, Elon Musk, the founder of Tesla, employment of Apple’s stylish computer system as an analog to conceive of his popular EV (Martins et al., 2015).

Extant literature has indicated that business practitioners and managers hold unique *images* of their operational environment (Massa et al., 2017) and even *network pictures* that depict their ways of developing inter-organizational relationships, in line with their individual sense-making of the surroundings, for the attainment of the economic goals (Ramos et al., 2012). This view of network pictures is grounded in the fact that any actors, both individuals and organizations, require to be engaged in interaction with others for their sustainable development (Håkansson et al., 2009), and that the actors’ actions and reactions are influenced by their own interaction histories taking place in relational bounds, within which perceptions and understandings are developed (Weick, 1995; Håkansson and Ford, 2002). This interactive perspective emphasizes that a firm’s competitive advantage hinges on its business interaction with others, by which the engaged actors’ resources can be combined and their activities can be systematically connected for productive uses, from which the creation and capturing of value are rendered (Baraldi et al., 2007). As a result, network pictures need to be regarded as a crucial part of managerial schemas because these mental pictures are concerned with key components in the design of a business model, including the boundary-spanning resources and activities that are contributed by important partners (Osterwalder and Pigneur, 2010; Zott and Amit, 2010). Moreover, the network picture-infused mental representations are particularly important to competing in the era of platforms and ecosystems (Adner, 2017; Jacobides et al., 2018), due to which the firm’s advantage relies on an adequate design of interdependencies that permit value creation in collective and aligned actions. Thus, it is important to have the flexibility to learn and improve the existing conceptual framework so that consequent actions and behavior can be elevated (Hall, 1976; Hall, 1984). Moreover, the persistence of improper conceptual framework does explain organizational decline as a protracted process or a downward spiral despite the abundance of managerial talent (Hambrick and D’Aveni, 1988).

Additionally, in the era of digital transformation, entrepreneurial firms usually confront tasks on how to utilize emerging technologies to perform cognitive automation (Lu et al., 2020; Edwards, 2021) as decision-making support systems to shape and execute their business models accurately and effectively. For example, applications of Internet-of-things (IoT) and artificial intelligence (AI) technologies can help entrepreneurial firms bring about cyber-physical smart manufacturing by automatically sensing, collecting, and analyzing data for decision-making through networking operations, equipment, factories, and personnel (Coatney, 2019; Jones et al., 2020; Durana et al., 2021; Edwards, 2021; Hamilton, 2021; Kovacova and Lăzăroiu, 2021) to enhance overall performance and swiftly adjust to ever-increasing end-user demands at a competitive cost (Keane et al., 2020; Monostori and Váncza, 2020; Popescu et al., 2021). That is a system of cyber-physical smart manufacturing tackles reflections from both the supply and demand sides by the regenerative consequences of dynamic effectiveness (Mikalef et al., 2020; Brown and Gu, 2021) through the use of a responsive self-governing process, which plays a significant role in formulating and implementing business models. In addition, the capability for entrepreneurial firms to robotically, precisely, and predictably validate process signatures and communicate the upgrade of production parameters leads to optimization in quality, time management, and consistent transparency throughout the entire value chain (Lenz et al., 2020; Watkins, 2021), which is crucial to ensure survival and avoid failure in their business operations.

## Research Methodology

### Qualitative Approach

Given the exploratory nature of this research, employing a qualitative naturalistic inquiry is an appropriate method (Lincoln and Guba, 1985), which enables us to study the research phenomena being embedded in socially constructed reality (Lincoln and Denzin, 2003). The benefits of employing such a discovery-oriented method include the reduction of study manipulation of the research setting and few prior restrictions on what the consequences of the investigation will be (Patton, 2002). To tackle our research question of how an entrepreneurial firm’s business model is influenced by its organizational members’ managerial cognition, we utilized a single case study to investigate the complexity and complicatedness of an entrepreneurial initiative (Yin, 2009), including the contextual conditions in its unique setting (Myers, 2019), within which the perceptions of involved actors were formed (Weick and Bougon, 1986). In particular, the case study allowed us to concentrate on the influences of time and temporality (Chou and Zolkiewski, 2012), which drove the developmental process of a business model underpinning an entrepreneurial initiative.

### Research Case Selection

The case under investigation was Better Place, the focal entrepreneurial company based in Israel, which was chosen under the following considerations. First, the chosen company served as a suitable case for engaging in an intellectual dialogue with business model literature (Yin, 2009), mainly because Shai Agassi, the founder of Better Place, conceived a revolutionary idea of promoting his proprietary electric cars by separating the ownership of car and battery and put it into business practice. Second, Better Place was selected because it was regarded by the media as the most spectacularly failed entrepreneurial start-up of the twenty first century, and thus serves as an archetypal example of entrepreneurial failure. Furthermore, this case permitted us to explore our research question because Better Place had a complete developmental process from its establishment in 2007 to its liquidation in 2013. Since Better Place was famous for its entrepreneurial initiative, an abundance of archival materials was available to build the case, including the entrepreneur’s speeches and interviews of its various stakeholders in the media. Therefore, using Better Place as the case is a suitable way to look at entrepreneurial failure from a theoretical lens of business models.

### Data Collection and Analysis

This research collected informative archival materials including the transcripts of speeches of the founder, individual, and group interviews in the media, press reports, and relevant seminar and conference materials. A variety of data sources and collection paths also allowed this research to examine questions from more perspectives. Secondary data of individual and group interviews with the media from 2007 to 2020 have been collected. This period covers the entire life of Better Place from its foundation to bankruptcy and several years later, as public opinion reacted. Table 1 lists individual and group interviews by 42 personnel, which resulted in a total of 65 h 30 min and 44,791 words in length, with 6 categories of interviewees ranging from the CEO of Better Place, company employees, partners, competitors, investors, and industry successors. In every interview, unstructured inquiries and open questions were used to understand the background and experiences of the interviewees as well as their reactions to certain issues. During this research, we have also actively participated in various formal and informal activities related to Better Place and the EV industry, including international seminars, and conferences hosted by industrial associations and businesses. Our participation in these activities has served two purposes; one is to collect related secondary data, and the other is to find more potential interviewees. More secondary data have also been collected from a total of 45 reports published by international media, periodicals, and industrial associations. These reports provide sufficient evidence for historical analysis.

**TABLE 1:** Categories of identities of the persons interviewed by media (2007–2020).

	Category	Number	Note	Length (h/words)	Media
Group interviews	Employees	5	High-level executives, mid-level management, staffs	3′00″ (h) 1,375 (words)	YouTube videos, Financial Times, Ynetnews
	Partners	8	EV manufacturers, vehicle leasing providers, governments, power companies	5′30″ (h) 2,583 (words)	YouTube videos, Green Car Reports, Forbes, Car Advise, The Wall Street Journal
	Competitors	5	Toyota, Autolib, Tesla	4′30″ (h) 1,898 (words)	YouTube videos, The Guardian, Forbes
	Industry successors	6	GM, BMW, Tesla, Renault-Nissan, GreenWay	2′00″ (h) 3,206 (words) 2,495 (words)	YouTube videos, TechCrunch, Forbes
	Sum	24		15′00″ (h)/ 11,572 (words)	
Individual interviews	CEO	1	Shai Agassi	32′30″ (h) 19,688 (words)	TED, YouTube videos, The Washington Post, The Atlantic, cnet.com, The Wall Street Journal
	Employees	3	High-level executives, mid-level management	5′32″ (h) 1,433 (words)	YouTube videos, Forbes, The Jerusalem Post
	Partners	3	EV manufacturers, vehicle leasing providers	4′30″ (h) 4,803 (words)	YouTube videos, The Times of Israel, Israel21c, Green Car Congress, The Wall Street Journal
	Competitors	2	Autolib, Tesla	3′50″ (h) 549 (words)	YouTube videos, Yahoo finance, the Atlantic
	Investors	7	General Electric, HSBC Holdings, morgan Stanley, European Investment Bank, UBS AG, VantagePoint, Ofer Group, Lazard Asset Management, Maniv Energy Capital	0′50″ (h) 4,309 (words)	YouTube videos, Greentech Media, CBS Money Watch
	Industry successors	2	Tesla, Renault-Nissan	3′18″ (h) 2,437 (words)	YouTube videos, Yahoo finance, New York Times, The Atlantic, Globes, Green Prophet, Reuters
	Sum	18		50′30″ (h) 33,219 (words)	
Total		42		65′30″ (h) 44,791 (words)	

To analyze the relevant case data, we relied on the technique of “segmenting and reassembling the data to convert them into findings” (Boeije, 2010). We employed the procedure suggested by Miles and Huberman (1984) that revealed data reduction, display, and conclusion verification that are interwoven before, during, and after data collection. Although data from different sources such as media channels do not show all perspectives in common, the utilization of this field-based dynamic research provides an advantageous complement to the data acquired and permits us to narrow the gap between existing data and the relational respects of facts.

We first proceeded with data reduction techniques, guided by the research question: how is an entrepreneurial firm’s business model influenced by its organizational members’ managerial cognition? We first coded all archival materials we obtained to indicate their source, e.g., speeches of the founder, individual and group interviews in the media, press reports, and seminar and conference materials. Then, we prepared a list to sort all issues reflected and involved in this study. Since the accurate interpretation of events requires an understanding of context, we developed a context table to map descriptions of data with the involved issues. Following that, we focused on issues deemed to have a greater effect on the business models and organizational members’ socio-cognition, and a total of 23 such issues were identified. A total of 17 issues were retained because they met critical criteria, while the remaining issues were eliminated for being relatively minor in comparison with the former.

Then, we continued our efforts to create a string of information displays to process the data, which contained a series of role-category matrices arrayed in temporal sequence. For each archival item, we produced a 5 × 10 matrix that featured the rows presenting the identities of the participants in the interviews and the columns showing the founder’s ideas and behavior, partners’ ideas, and behavior, the company’s strategy and major events, customers’ reactions and behavior, and the outcome of the strategy and major events. Then, the main points of various individuals’ comments regarding the selected issues were input into each cell, grouped by role categories. We accordingly connected and coded related comments in time sequence to observe the effects. As a consequence, we developed 10 of these role-category matrices arrayed by time series.

Finally, the finished matrices were integrated to build a summarized 5 × 7 matrix (Table 2) with the rows showing each year from 2007 to 2013 and the columns presenting the founder’s ideas and behavior, stakeholders’ ideas and behavior, the company’s strategy and major events, customers’ reactions and behavior, and the outcome of the strategy and major events. Information in each cell marked the major events identified from the previous matrices according to the perceptions of interviewees from individual and group interviews. This display allowed us to trace the evidence of how an entrepreneurial firm’s business model is affected by its organizational members’ socio-cognition around all major events.

**TABLE 2:** Better Place’s time line of issues and events.

When the company’s major events started to occur	Founder’s ideas and behavior	Stakeholders’ ideas and behavior	Company’s strategy and major events	Customers’ reactions and behavior	Outcome of the strategy and major events
2013	The founder stepped down with little influence on the company as one of the corporate board members.	The only partner, Renault-Nissan, saw a dim future for swap-battery EVs and stopped further collaboration with Better place.	1. CEO Evan Thornley stepped down, replaced by Cohen. 2. Withdrew from markets of the U.S., Australia, Japan, and China, and only focused on markets of Israel and Demark.	Customer confidence was severely crushed.	1. The financial status of the company deteriorated. 2.Better Place announced bankruptcy.
2012	Lack of capability for execution.	President Ofer of Better Place’s company in Israel disagreed with the founder agassi regarding future markets.	Founder Shai Agassi was requested to step down from CEO position (was still corporate board member), replacing by the CEO of Better Place’s branch company in Australia, Evan Thornley.	Lack of will to purchase an EV with doubtful resale value, an infantile network of swap stations, and an inexperienced contract for battery power.	1. Better Place failed to develop a growing EV customer base or create effective strategic partnerships. 2. Better Place was facing a financial dilemma.
2011	Sought to expand the market of Guangzhou City, Canton Province, China.	1. Government of China listed EV as a promising strategic industry. 2. China Southern Power Grid (CSG) and Better Place signed a partnership MOU to form alliance.	Sought to expand the market of Guangzhou City, Canto Province, China.	Lack of will to purchase an EV with doubtful resale value, an infantile network of swap stations, and an inexperienced contract for battery power.	Slow review by local authorities and EV manufacturing problems hindered the market expansion plan.
2010	Sought to expand Australia market.	An alliance was formed between ActewAGL (Australia’s power company) to expand the Australia market.	Sought to expand Australia market.	Consumers hesitate to purchase due to potential risks derived from innovation of EVs.	Slow review by local authorities and EV manufacturing problems hindered the market expansion plan.
2009	1. Sought to expand the market of Province of Ontario, Canada. 2. Sought to expand the market of Japan. 3. Tends to employ cronies as top management of the company.	1. An alliance was formed between Bullfrog (a power company in Canada) and Better Place to expand Canada market. 2. Government of Japan supported the setting up of EV charging stations in Japan; an alliance was formed between Nihon Kotsu (largest taxi operator in Japan) and Better Place. 3. Many high-level executive managers of Better Place left the company.	1. Sought to expand the market of the Province of Ontario, Canada. 2. Set up first EV charging station in Japan.	General customers still regarded EV battery swapping and charging as a relatively new technology.	1–2. Slow review of local authorities and EV manufacturing problems hindered the market expansion plan.
2008	1. Sought to expand the Israel market. 2. Sought to expand the Demark market. 3. Sought to expand the market of Bay Area, State of California, United States. 4. Sought to expand the market of the State of Hawaii, United States. 5. Sought to deploy battery swap system and declared that cost would be half the price of a model petroleum fueling station. 6. Sought to use smart software to supervise and manage the EV recharging system; sought to encourage governments to mandate access and to charging networks to boost competition. 7. Was unrealistic, stuck to his own opinions, unwilling to compromise when negotiating with potential partners.	1. Government of Israel lowered the EV sales tax. 2. Dong Energy provided funding to expand Demark market. 3. 4. Governments of Hawaii and California facilitated market development. 5. Only Renault but no other carmakers joined Better Place’s big plan. 6. Other potential partners, such as leasing companies, were unwilling to invest large capital into an asset with unclear value. 7. The business models were still so novel that potential partners (car makers, battery manufacturers, swap/charging station manufacturers, power companies, and local governments) hesitated to engage.	1. Sought to expand the Israel market. 2. Sought to expand the Demark market. 3. Sought to expand markets in the Bay Area, State of California, United States. 4. Sought to expand the market of the State of Hawaii, United States. 5. Launched the QuickDrop battery swap system to support the Renault Fluence Z.E. battery. 6. Launched its first functional charging station in Israel. 7. In the process of negotiation with potential partners.	1. The Better Place business model received a lukewarm response from the automotive press. 2. General customers still regarded EV battery swapping and charging as a relatively new technology. 3. Corporate customers hesitated to purchase the EV car because even though the purchase cost declined (due to the reduction of purchase tax), the cost of possession still increased (license tax includes battery usage fee).	1-4. Slow review of local authorities and EV manufacturing problems hindered the market expansion plan. 5.Robotic swap stations were supposed to cost $500,000 each, but ended up costing $2 million. 6.Lack of global consensus for governments to mandate international standards and access to recharging networks.
2007	1. EV dream: customers buy the cars while Better Place owns the batteries. 2. Emphasis on infrastructure creation rather than value creation.	Began with strong financial and non-financial support from Israel’s President Peres, Renault-Nissan, Garnoff Venture, Maniv Energy Capital, Morgan Stanley, and Vantage Point.	Better Place was founded and venture-backed as an international company with business models to develop and sell battery swap and charging services for EVs.	1. Venture investors were interested in the new business models. 2. Consumers felt the idea was novel and started to evaluate the business model.	Acquired financial investment successfully from venture capitals.

## Findings and Analysis

### Better Place’s Business Model and Environmental Context

The Israel-based Better Place, founded in 2007, was a venture-backed international firm that developed and rendered battery swap and charging services for EVs. Its founder and CEO at the time, Silicon Valley entrepreneur Shai Agassi, pledged to change the world by means of service stations where a battery in an EV could be taken out and swapped for a fresh one, extending the EV’s driving range within minutes. Agassi’s ambition had at first been supported by Israel’s President Peres. The Israeli government at that time made commitments to provide government administrative incentives and had also been backed up by one carmaker, Renault-Nissan (which then became Better Place’s critical partner in EV manufacturing), as well as certain famous venture capital groups such as Garnoff Venture (was responsible for Better Place’s public relations), Maniv Energy Capital, Morgan Stanley, and Vantage Point (which provided strong funding). The value proposition of Better Place, which shaped and formulated the company’s business model, was as Agassi described to the media as follows: “*Customers would buy the cars, but Better Place would own the batteries.*”^1^ That meant when drivers needed to recharge their EVs speedily, they could proceed to a Better Place station and easily swap their batteries within minutes.

In Agassi’s plan, by employing automation software to supervise and manage the recharging stations connected with Better Place, the company would be able to offer electricity for numerous EVs without increasing any power plants or transmission lines. In addition, Better Place’s first prototype car was the Renault Fluence Z.E., an electric sedan equipped with a swappable battery in its trunk space that could provide an 80-mile driving range. Better Place’s QuickDrop battery exchanging system allowed the battery of Renault Fluence, the only EV disposed of in Better Place’s network, to be switched in merely 3–5 min at battery swap stations.

Furthermore, Better Place established its first EV charging station in December 2008 in Cinema City, Israel. The company’s network of EV charging infrastructure was based on a cutting-edge smart grid platform that could automatically manage the charging of hundreds of thousands of EVs simultaneously during daily peak demand hours and prevent the network from overloading in the host country. Better Place also encouraged governments to mandate open access and international standards to EV charging stations so as to facilitate competing networks. However, sufficient global consensus in support of this proposal was not achieved.

Although Better Place successfully gained seed funding from venture capitals when it was founded, the initial enthusiasm did not last. Better Place’s EV battery swap and charging solutions were competing with many other technologies, all of which were targeting to timely address the most imminent energy problems then, most importantly decarbonization. The industry in which it operated, no matter whether renewable energy or EV, was full of emerging markets where applications of new technologies arose rapidly. Examples included biofuels, wave, tidal, solar energy, thermal storage, smart meters, gas storage, onshore and offshore wind, and hydrogen vehicles. They were so innovative and novel in the industry that the prospects were still hazy and unexpected. There was much disagreement, and consensus failed to emerge regarding which application would prevail in the foreseen future. From a financial perspective, EV battery swap and charging was progressing up the funding maturity curve but remained a relatively emergent technology. That foreshadowed the subsequent difficulties Better Place would face in funding acquisition once they could not make obvious progress with partnership or market feedback.

As for partnership building, as an EV charge point operator, its various potential partnership options, in terms of charging segments, included home, destination, rapid, and workplace charging. For example, in the case of home charging, its partnership options included EV manufacturers, vehicle leasing providers, housebuilders, councils, retail energy suppliers, and renewable energy hardware (such as solar PV and battery storage). For the funding of any go-to-market solutions, they needed to declare a solid and reliable revenue model. Creating profit based on a growing EV customer group was crucial, and strategic partnerships were a key tool to help deliver the growth. However, Better Place neither succeeded in developing a growing EV customer base nor did it create effective strategic partnerships.

A bold attempt in an entrepreneurial initiative but mistaken sense and decision-making amid environmental changes and competition led to an eventual financial crisis at Better Place. A report stated as follows: “*While each swap station would cost $500 thousand, the then CEO of Better Place, Shai Agassi, declared that the cost would be half the price of a typical petroleum fueling station*…*and it ended up costing $2 million. Critically, Better Place failed to get any other automaker onboard to design and produce standardized vehicles with swappable batteries, with Agassi alienating such potential partners as BMW and GM. Better Place sold fewer than 1,500 electric Renaults before it was liquidated, with Agassi fired in disgrace in 2012.*”^2^

Better Place after all filed for bankruptcy in May 2013. After spending approximately US$850 million in private capital, the company underwent two failed attempts at post-bankruptcy acquisition. In the end, the bankruptcy receivers sold off the last assets to Gnrgy for only US$450,000 in November 2013.

### The Interrelation of Better Place’s Organizational Members’ Managerial Cognition and Its Business Model Performance

Based on Table 2, it can be observed that the company’s major events, usually derived from strategic changes in its business model, were influenced by its founder Agassi, the management of the company, and its various stakeholders. They are jointly interrelated to influence the firm’s decision-making on business model development, which is critical to company performance.

First, Table 2 shows that the founder Agassi has a tendency to be arbitrary, unwilling to compromise, narcissistic, inclined to employ cronies as top management of the company, and lacked execution capability. These features triggered his epochal EV dream that customers would purchase the EVs, but Better Place would own the batteries, his emphasis on infrastructure creation rather than value creation, and his ambition to expand into overseas markets such as Israel, Demark, the United States, Canada, Japan, Australia, and China. All the ideas and behavior of the founder impacted the company’s business model and major events.

Second, we also find from Table 2 that in many major events of the company, the founder was not the only actor whose ideas and behavior could impact the company’s decision-making on the business model. The members from its top management and the other stakeholders among its partners, such as EV manufacturers, investors, leasing companies, and local governments, were also important influencers that did much to determine how Better Place would run its business. Their joining even brought new teams and organizational members to Better Place, which could directly carry significant weight concerning the development of the company’s business model. For instance, as Better Place was founded in 2007, although the vision of the founder Agassi played a decisive role, various venture capitals and Israeli President Peres were also important driving forces that encouraged the company to go for its great EV dream. Similarly, after Better Place launched operations, when President Ofer of Better Place Israel disagreed with the founder Agassi regarding future markets and the only partner Renault-Nissan took a dim view of the future prospects of swap-battery EVs and stopped further collaboration with the company, Better Place took actions to withdraw from markets in the United States, Australia, Japan, and China, and chose to focus only on the markets of Israel and Demark.

Third, based on the above, we can figure out that when Better Place was first founded, the views and positions of its influential decision-makers, including the founder and the other stakeholders, were very consistent, which did create a great impetus to move the business forward. However, shortly after it launched operations, management missteps and the failure of Better Place to convince its business partners of its business model led to fragmentation among decision-makers, who had divergent interpretations of company’s strategies and business models. This caused Better Place’s gradual decline. Clues regarding how Better Place’s initial problems resulted from management missteps were also revealed in a report as follows: “*Mr. Agassi focused too broadly on future growth, at the expense of getting early details of the business right.*” and *“As Better Place expanded, Mr. Agassi recruited many software specialists*… *but the company had fewer managers with automotive or infrastructure expertise.*”^3^

Fragmentation among decision-makers also brought about misjudgment of environmental and market risks. Although a business model focused on a battery-swap station network made the company unique, fascinating, and compelling, unfortunately, each station cost around US$500,000 to construct. It was unwise to develop an enormous network for merely few users. Without building a complete network, the customers were reluctant to adopt Better Place’s industry-leading idea of battery swap for EVs. The chicken-and-egg conundrum can be time and cost-consuming to overcome and perhaps was not even necessary. This just explained why Renault, a well-known French brand, only sold 800 EVs that adopted Better Place’s innovative solution in the initial period.

Moreover, an innovator needs a solid foothold to survive in markets. It counts on customer groups willing to adopt the new technology rapidly in the early stage. Better Place had trouble in identifying the foothold it was targeting. The company began with the goal of exploring the markets of Israel, Denmark, the United States, and Australia, but ended up by trimming its ambitions to only Israel and Denmark. Weak support from local authorities also hindered its market expansion plan, which could be seen in an interview of the company officials, where one person stated as follows: “*Local authorities, whose permission was needed to build battery-switching stations, put up unexpected roadblocks, slowing progress.*”^4^ Contrast Better Place’s approach to that of its French competitor, Autolib, which has established a network of presence throughout Paris where a proprietary EV (the “Bluecar”) could be rented by early adopters by the hour. Its strategic focus was so local that the charging station network was sufficient.

In addition, in new markets, the early adoption rate is correlated directly with the extent of risk that customers are taking. Better Place forced consumers to buy an EV with the doubtful resale value, or to lease one from leasing firms assumed to be willing to take the cost of the asset. Furthermore, the company also locked people into purchasing expensive charging stations and using an infantile network. All these resulted in its failure in market penetration. If we contrast Better Place’s approach with the way Nissan leased its Leaf EV or the reason why Tesla’s EVs required no dependence on charging locations, it is not difficult to find that innovation is rarely so compelling that customers would not refuse to be among those of the early adopters. The Apple’s continuous release of its successful new products, including the iTunes, iPhone, and iPad, has taught us a valuable lesson that the leader or management team of a startup needs to prioritize a decision that could generate customers’ sustaining dependence on the firm’s production solution, followed by a lock-in effect.

It is important to note that revolutionary ideas gain influence if they are realized through a simple product design. However, Better Place was not the case. In the eyes of its founder, peddling Better Place’s unique business model seemed to be more important than promoting its vehicles that generated actual incomes. The company’s efforts resulted in a lukewarm response from the automotive press. It turned out to become that the innovative seemingly, but yet doubtful offering, in terms of a mediocre vehicle together with an additional contract for electricity, made it hesitate for customers to buy-in. One report interpreted the failure of Better Place as follows: “*The execution of going to market was faulty on several fronts, including failure to fully develop and validate the system in the launch city of Tel Aviv, an aggressive international expansion without having proven the model, and failure to find an effective way of communicating the value proposition to a substantial base of potential subscribers. These are all issues that could have been remedied, however, had financing not been a constraint.*”^5^

## Discussion and Implications

### Discussion

Our case findings show that an entrepreneurial firm’s business model demonstrates how the firm operates in a certain institutional environment (Magretta, 2002) and is interpreted by organizational members (Ring and Rands, 1989; Massa et al., 2017). How organizational members’ managerial cognition influences an entrepreneurial firm’s business model within this research context can be illustrated by the interrelation of the managerial cognition of Better Place’s organizational members and its business model performance. The founder Agassi’s personal character, emotions, attitudes, and the capability of sense-making, together with the managerial cognition of Better Place’s organizational members, including its top management, partners, investors, and the other influential stakeholders, were inextricably intertwined as a conceptual framework (Massa et al., 2017) that formatted Better Place’s business model. Therefore, an entrepreneurial firm’s business model is determined not merely by its entrepreneur but is also influenced by its organizational members, including various stakeholders, based on their individual understanding, knowledge, and experience (Weick, 1995) and thus, their decision-making and strategic actions are conditioned by their interpretations of the world around them (Gavetti and Rivkin, 2007). Enterprises can hardly have business models designed on their own. The business model of an enterprise is closely connected with that of its stakeholders Mason and Spring (2011). Organizational members, including stakeholders, with their mind representations and conceptual framework, usually employ existing knowledge, abstractions, and concepts from a familiar area and apply them to a new area to make sense of novelty and ideate important elements for developing a business model (Martins et al., 2015). A firm’s competitive advantage hinges on its business interaction with others, by which the engaged actors’ resources can be combined, and their activities can be systematically connected for productive uses, from which the creation and capturing of value are rendered (Baraldi et al., 2007).

In Better Place’s case, as the company’s stakeholders, such as its partners, investors, and local governments, increased, they also brought in new members and teams with new expertise, habits, and motivations to the company and added new knowledge to the conceptual framework, which critically commanded the decision-making on the structuring, formation, and execution of its business model. The empirical evidence in this research can be found from the influence of Renault-Nissan, Better Place’s only car-maker partner. Renault-Nissan came to have a dim view of the future prospects for swap-battery EVs, which caused Better Place to withdraw from a number of foreign markets and reshape its business model.

In addition, owing to the founder Agassi’s idealistic character, fragmentation among Better Place’s decision-makers, and the breakdown of the conceptual framework shortly after its foundation, it failed to convince its internal members as well as broad partners. As a result, Renault was the only carmaker that joined the big plan. Weak support from local authorities also hindered its market expansion plan. Those factors eventually caused Better Place’s crash. Moreover, the business model derived from its conceptual framework (in other cases, such as Nissan and Tesla, their cognition actually guided them to different business models) forced consumers to buy an EV with the doubtful resale value, lease one from leasing firms assumed willing to take the cost of the asset, locked people into purchasing expensive charging stations or using an infantile network of swap stations, causing the failure of the company to develop effective narratives in communication with its internal members, broad partners, investors, and customers, and lead to eventual failure. This proves the importance of business models as schemas organizing managerial understanding of how firms’ value-creating activity systems are designed. Organizational members usually not only consider the cognitive aspect (collective and individual) but also the linguistic one (communicating within the organization) (Brown et al., 2005; Martins et al., 2015). This provides empirical evidence that the persistence of improper mental models does explain organizational decline as a protracted process or a downward spiral despite the abundance of managerial talent (Hambrick and D’Aveni, 1988), and it is important to have the flexibility to learn and improve existing mental models so that consequent behavior and actions can be elevated (Hall, 1976; Hall, 1984).

### Theoretical Implications

Based on the discussion of our case findings, this research puts forward the following propositions:

Proposition 1: Organizational members’ managerial-cognition-oriented conceptual framework based on their interpretations of environments is critical to the business model decision-making of an entrepreneurial firm.

Proposition 2: Discrepant and strongly held conceptual frameworks of an entrepreneurial firm tend to result in misjudgment of environmental changes and cause inappropriate decision-making on its business model.

Proposition 3: Inappropriate decisions are easily made on a business model, especially when emerging-market numbers in an industry are high and consensus regarding technology innovation in an industry is still lacking.

### Practical Implications

This research can also provide practical implications for the development of entrepreneurial businesses. In Better Place’s case, the company was facing a complex environment with numerous emerging markets where various innovative technologies seeking decarbonization competed. In the beginning under the founder Agassi’s cognition and plan, with the early support from its most stakeholders, it aimed to penetrate the markets of Israel, Denmark, the United States, and Australia. However, owing to a series of subsequent misjudgments of market changes and mistaken decisions on its business models, the company eventually trimmed its ambitions to Israel and Denmark, causing its failure to acquire a firm foothold. Local authorities’ unexpected delay in the review also hindered its market expansion plan. At that time, within the industry of either renewable energy or EV, so many novel ideas and business models emerged and competed with each other that consensus regarding technology innovation had been hard to reach. Therefore, we also found that the conceptual framework of an entrepreneurial firm tends to lead to mistaken decisions on business models, especially when emerging-market numbers in an industry are high, and consensus regarding technology innovation in an industry is still lacking. These findings can be viewed as reminders for entrepreneurial businesses to operate cautiously and think carefully when making critical decisions. For instance, savvy entrepreneurial firms may be well advised to take slower steps and not do dramatic experiments with large budgets at the initiative stage of their businesses. Instead, a small pilot, a dry run of a new technique, or a simulation for markets, may be more dependable and secure.

Moreover, if entrepreneurial initiatives are to avoid failure, it is crucial that they have the flexibility to learn and improve existing mental models and consequent business models. Entrepreneurial firms can utilize innovative technologies, such as IoT and artificial intelligence, to facilitate their dynamic work on improving shared mental models through cognition automation. The result of this research can also provide a useful foundation for entrepreneurial firms in constructing machine learning systems to autosense and maintain the optimal conceptual framework to avoid failures and ensure survival.

## Conclusion and Future Research

This research pioneers by explaining the failure of an entrepreneurial initiative through a holistic lens by integrating business models and a managerial cognition perspective. We comprehensively and logically provide clarity on the phenomenon, including the fact that organizational members’ managerial cognition and conceptual framework based on their interpretations of environments are critical to the decision-making on the business architecture in an entrepreneurial firm. The discrepant and strongly held conceptual framework may result in misjudging the environmental changes, especially when emerging-market numbers in an industry are high, and consensus regarding technology innovation in an industry is still lacking. The improper conceptual framework can generate mistaken business models, which further bring about an organizational decline. If entrepreneurial initiatives are to avoid failure, it is crucial that they have the flexibility to learn and improve existing conceptual frameworks and consequent business models.

The result of this research provides value both theoretically and practically. Theoretically, the contribution of this research to the study streams of entrepreneurship is that the use of a managerial cognition perspective that focuses on managers’ mental representations, recognized as schemas (Massa et al., 2017), enables us to provide a deep understanding of the failure of an entrepreneurial business model, complementing the knowledge of business models that take from a rational design and an evolutionary learning perspective (Chesbrough, 2010; Zott and Amit, 2010). Practically, the outcome of this research can also be viewed as a reminder for entrepreneurs and practitioners to operate cautiously and think carefully in handling decision-making at initiative stages of business. Entrepreneurial firms that can catch, correct, and learn from failure before others do usually succeed.

This research faces constraints as other studies usually do. First, it is limited to inference only from a single case study, especially under the circumstance that failure in entrepreneurship is pervasive. Second, since Better Place had gone bankrupt in 2013, it also caused difficulty in gathering primary data. However, due to the long engagement of one of these research authors in the case of Better Place, the abundance of existing evidence from archival data still provide a solid research foundation. During the research process, triangulation was conducted as well, including examining the variety of data resources and the challenges and inquiries from the coauthor.

The propositions developed by this study still need more empirical tests on multiple cases in order to form theories. Future research, whether qualitative or quantitative, is encouraged to go further based on the outcome of this research. Furthermore, learning from failure paves the way to success. In the era of digital transformation, the result of this research can also serve as a foundation for future empirical studies contributing to machine learning of entrepreneurial firms with regard to such fields as cognitive automation, artificial intelligence-driven manufacturing, and sustainable and industrial value creation.

## Data Availability Statement

The original contributions presented in this study are included in the article/supplementary material, further inquiries can be directed to the corresponding author.

## Author Contributions

M-HL and H-HC contributed to the conception and design of the research. M-HL organized the structure, compiled the theoretical background, and wrote the first draft of the manuscript. H-HC collected the data, refined the logic analysis, and examined the interpretations. Both authors contributed to the manuscript revision and approved the submitted version.

## Conflict of Interest

The authors declare that the research was conducted in the absence of any commercial or financial relationships that could be construed as a potential conflict of interest.

## Publisher’s Note

All claims expressed in this article are solely those of the authors and do not necessarily represent those of their affiliated organizations, or those of the publisher, the editors and the reviewers. Any product that may be evaluated in this article, or claim that may be made by its manufacturer, is not guaranteed or endorsed by the publisher.

## Footnotes

1 The Washington Post, 6 March 2013. Refer to https://www.washingtonpost.com/news/wonk/wp/2013/03/06/better-place-was-supposed-to-revolutionize-electric-cars-what-went-wrong/.

2 Lawrence Ulrich, Car Commentator, the IEEE Spectrum’s Blog, 13 May 2021. Available at https://flipboard.com/@ieeespectrum/tech-news-reqm0ejaz/how-is-this-a-good-idea-ev-battery-swapping/a-vqoALEffTAS4cm4KrXlrbA%3Aa%3A1880309793-1cdfd047f6%2Fieee.org.

3 Interviews of former employees of Better Place, 2013. Refer to https://www.washingtonpost.com/news/wonk/wp/2013/03/06/better-place-was-supposed-to-revolutionize-electric-cars-what-went-wrong/.

4 Interviews with company officials, The Washington Post, 6 March 2013.

5 Harvard Business School Digital Initiative, 10 December 2015.
